# Transmission of SARS-CoV-2 on mink farms between humans and mink and back to humans

**DOI:** 10.1126/science.abe5901

**Published:** 2020-11-10

**Authors:** Bas B. Oude Munnink, Reina S. Sikkema, David F. Nieuwenhuijse, Robert Jan Molenaar, Emmanuelle Munger, Richard Molenkamp, Arco van der Spek, Paulien Tolsma, Ariene Rietveld, Miranda Brouwer, Noortje Bouwmeester-Vincken, Frank Harders, Renate Hakze-van der Honing, Marjolein C. A. Wegdam-Blans, Ruth J. Bouwstra, Corine GeurtsvanKessel, Annemiek A. van der Eijk, Francisca C. Velkers, Lidwien A. M. Smit, Arjan Stegeman, Wim H. M. van der Poel, Marion P. G. Koopmans

**Affiliations:** 1 Department of Viroscience, Erasmus MC, WHO Collaborating Centre for Arbovirus and Viral Hemorrhagic Fever Reference and Research, Rotterdam, Netherlands.; 2 Royal GD, Deventer, Netherlands.; 3 Netherlands Food and Consumer Product Safety Authority (NVWA), Utrecht, Netherlands.; 4 Municipal Health Services GGD Brabant-Zuidoost, Eindhoven, Netherlands.; 5 Municipal Health Services GGD Hart voor Brabant, ‘s-Hertogenbosch, Netherlands.; 6 Municipal Health Services GGD Limburg-Noord, Venlo, Netherlands.; 7 Wageningen Bioveterinary Research, Lelystad, Netherlands.; 8 Stichting PAMM, Veldhoven, Netherlands.; 9 Division of Farm Animal Health, Department of Population Health Sciences, Utrecht University, Utrecht, Netherlands.; 10 Institute for Risk Assessment Sciences (IRAS), Utrecht University, Utrecht, Netherlands.

## Abstract

Animal experiments have shown that nonhuman primates, cats, ferrets, hamsters, rabbits, and bats can be infected by severe acute respiratory syndrome coronavirus 2 (SARS-CoV-2). In addition, SARS-CoV-2 RNA has been detected in felids, mink, and dogs in the field. Here, we describe an in-depth investigation using whole-genome sequencing of outbreaks on 16 mink farms and the humans living or working on these farms. We conclude that the virus was initially introduced by humans and has since evolved, most likely reflecting widespread circulation among mink in the beginning of the infection period, several weeks before detection. Despite enhanced biosecurity, early warning surveillance, and immediate culling of animals in affected farms, transmission occurred between mink farms in three large transmission clusters with unknown modes of transmission. Of the tested mink farm residents, employees, and/or individuals with whom they had been in contact, 68% had evidence of SARS-CoV-2 infection. Individuals for which whole genomes were available were shown to have been infected with strains with an animal sequence signature, providing evidence of animal-to-human transmission of SARS-CoV-2 within mink farms.

## Two-way transmission on mink farms

Severe acute respiratory syndrome coronavirus 2 (SARS-CoV-2) is a zoonotic virus—one that spilled over from another species to infect and transmit among humans. We know that humans can infect other animals with SARS-CoV-2, such as domestic cats and even tigers in zoos. Oude Munnink *et al.* used whole-genome sequencing to show that SARS-CoV-2 infections were rife among mink farms in the southeastern Netherlands, all of which are destined to be closed by March 2021 (see the Perspective by Zhou and Shi). Toward the end of June 2020, 68% of mink farm workers tested positive for the virus or had antibodies to SARS-CoV-2. These large clusters of infection were initiated by human COVID-19 cases with viruses that bear the D614G mutation. Sequencing has subsequently shown that mink-to-human transmission also occurred. More work must be done to understand whether there is a risk that mustelids may become a reservoir for SARS-CoV-2.

*Science*, this issue p. 172; see also p. 120

## Teaser

Genetic evidence indicates that SARS-CoV-2 transmission occurred from humans to mink as well as from mink to humans on farms in the Netherlands.

In late December 2019, severe acute respiratory syndrome coronavirus 2 (SARS-CoV-2) was identified as the cause of a viral pneumonia outbreak, possibly related to a seafood and live animal market in Wuhan, China (*1*). Since then, SARS-CoV-2 has spread across the world. By 8 October 2020, more than 36.1 million people had been infected with SARS-CoV-2, resulting in more than 1 million deaths (*2*). In the Netherlands, more than 155,000 infections have been confirmed, more than 6500 SARS-CoV-2–related deaths have been reported, and nonpharmaceutical interventions have been put into place to prevent further spread of SARS-CoV-2 (*3*).

In view of the similarities of the new virus and SARS-CoV-1, a zoonotic origin of the outbreak was suspected and linked to a Wuhan fresh market where various animals—including fish, shellfish, poultry, wild birds, and exotic animals—were sold. However, other cases with onset well before the period correlated with the Wuhan market–associated cluster were observed, which suggests the possibility of other sources (*4*). Although closely related coronaviruses found in bats (*5*, *6*) and pangolins (*7*, *8*) have the greatest sequence identity to SARS-CoV-2, the most likely divergence of SARS-CoV-2 from the most closely related bat sequence is estimated to have occurred between 1948 and 1982 (*9*). Therefore, the animal reservoir(s) of SARS-CoV-2 is(are) yet to be identified.

Similarly to SARS-CoV-1, SARS-CoV-2 binds to the host angiotensin-converting enzyme 2 (ACE2) receptor. On the basis of ACE2 similarities, a range of different animals have been used as models. Experimental infections in dogs (*10*), cats (*10*–*13*), ferrets (*10*, *14*), hamsters (*15*, *16*), rhesus macaques (*17*), tree shrews (*18*), cynomolgus macaques (*19*), African green monkeys (*20*), common marmosets (*21*), rabbits (*22*), and fruit bats (*23*) have shown that these species are susceptible to SARS-CoV-2, and experimentally infected cats, tree shrews, hamsters, and ferrets could also transmit the virus. By contrast, experimental infection of pigs and several poultry species with SARS-CoV-2 proved to be unsuccessful (*10*, *23*, *24*). SARS-CoV-2 has also sporadically been identified in naturally infected animals. In the United States and Hong Kong, SARS-CoV-2 RNA has been detected in dogs (*25*). In the Netherlands, France, Hong Kong, Belgium, Spain, and the United States, cats have tested positive for SARS-CoV-2 by reverse transcription polymerase chain reaction (RT-PCR) (*26*–*30*). Furthermore, SARS-CoV-2 has been detected in four tigers and three lions in a zoo in New York (*31*). In Italy, the Netherlands, and Wuhan, China, antibodies to SARS-CoV-2 have been detected in cats (*29*, *32*, *33*). Recently, SARS-CoV-2 was detected in farmed mink (*Neovison vison*), resulting in signs of respiratory disease and increased mortality (*29*, *34*).

In response to the outbreaks in mink farms,the Dutch national response system for zoonotic diseases was activated. Although the public health risk of exposure to animals with SARS-CoV-2 was determined to be low, increased awareness of animals’ possible involvement in the COVID-19 epidemic was needed. Therefore, from 20 May 2020 onward, mink farmers, veterinarians, and laboratories were obliged to report symptoms in mink (family Mustelidae) to the Netherlands Food and Consumer Product Safety Authority (NFCPSA), and an extensive surveillance system was established (*35*).

Whole-genome sequencing (WGS) can be used to monitor the emergence and spread of pathogens (*36*–*39*). As part of the surveillance effort in the Netherlands, more than 1750 SARS-CoV-2 viruses have been sequenced to date from patients from different parts of the country (*40*). Here, we describe an in-depth investigation into the SARS-CoV-2 outbreak in mink farms and mink farm employees in the Netherlands, combining epidemiological information, surveillance data, and WGS on the human–animal interface.

SARS-CoV-2 was first diagnosed on two mink farms (designated NB1 and NB2) in the Netherlands on 23 and 25 April 2020, respectively. After the initial detection of SARS-CoV-2 on these farms, a thorough investigation was initiated to identify potential transmission routes and to perform an environmental and occupational risk assessment. Here, we describe the results of the investigation of the first 16 SARS-CoV-2–affected mink farms by combining SARS-CoV-2 diagnostics, WGS, and in-depth interviews.

Owners and employees of the 16 mink farms with SARS-CoV-2–positive animals were included in the contact tracing investigation by the Dutch Municipal Health Services and were tested according to national protocol. Ninety-seven individuals were tested by either serological assays and/or RT-PCR. Forty-three of 88 (49%) upper respiratory tract samples tested positive by RT-PCR, whereas 38 of 75 (51%) serum samples tested positive for SARS-CoV-2–specific antibodies. In total, 66 of 97 (68%) tested individuals had evidence for SARS-CoV-2 infection (Table 1).

**Table 1:** Overview of human sampling on SARS-CoV-2–affected mink farms.

**Farm**	**First diagnosis** **in animals**	**Date(s) of sampling** **of employees and** **family members**	**PCR-positive** **individuals/tested** **individuals (%)**	**Serology-positive** **individuals/tested** **individuals (%)**	**Positive employees and** **family members/tested** **individuals (PCR and/or serology)**
NB1	23 April 2020	28 April 2020 to 11 May 2020	5/6 (83%)	5/5 (100%)	6/6 (100%)
NB2	25 April 2020	31 March 2020 to 30 April 2020	1/2 (50%)	7/8 (88%)	7/8 (88%)
NB3	7 May 2020	11 May 2020 to 26 May 2020	5/7 (71%)	0/6 (0%)*	5/7 (71%)
NB4	7 May 2020	8 May 2020	1/3 (33%)	2/2 (100%)	2/3 (66%)
NB5	31 May 2020	1 June 2020	2/7 (29%)	3/6 (50%)	3/7 (43%)
NB6	31 May 2020	1 June 2020	1/6 (17%)	4/6 (66%)	4/6 (66%)
NB7	31 May 2020	10 June 2020 to 1 July 2020	8/10 (80%)	NA†	8/10 (80%)
NB8	2 June 2020	3 June 2020	5/10 (50%)	5/9 (56%)	8/10 (80%)
NB9	4 June 2020	7 June 2020	1/7 (14%)	1/7 (14%)	2/7 (29%)
NB10	8 June 2020	11 June 2020	1/8 (13%)	3/8 (38%)	4/8 (50%)
NB11	8 June 2020	11 June 2020	1/3 (33%)	0/2 (0%)	1/3 (33%)
NB12	9 June 2020	11 June 2020	6/9 (66%)	2/8 (25%)	7/9 (78%)
NB13	14 June 2020	11 June 2020 to 18 June 2020	3/3 (100%)	0/2 (0%)	3/3 (100%)
NB14	14 June 2020	14 June 2020	1/3 (33%)	5/6 (83%)	5/6 (83%)
NB15	21 June 2020	10 June 2020 to 30 June 2020	2/2 (100%)	NA†	2/2 (100%)
NB16	21 June 2020	23 June 2020	0/2 (0%)	NA†	0/2 (0%)
Total			43/88 (49%)	37/75 (49%)	66/97 (68%)

*Serology was performed ~1 week before the positive PCR test.

†No serology was performed (NA, not applicable).

**Fig. 1:**
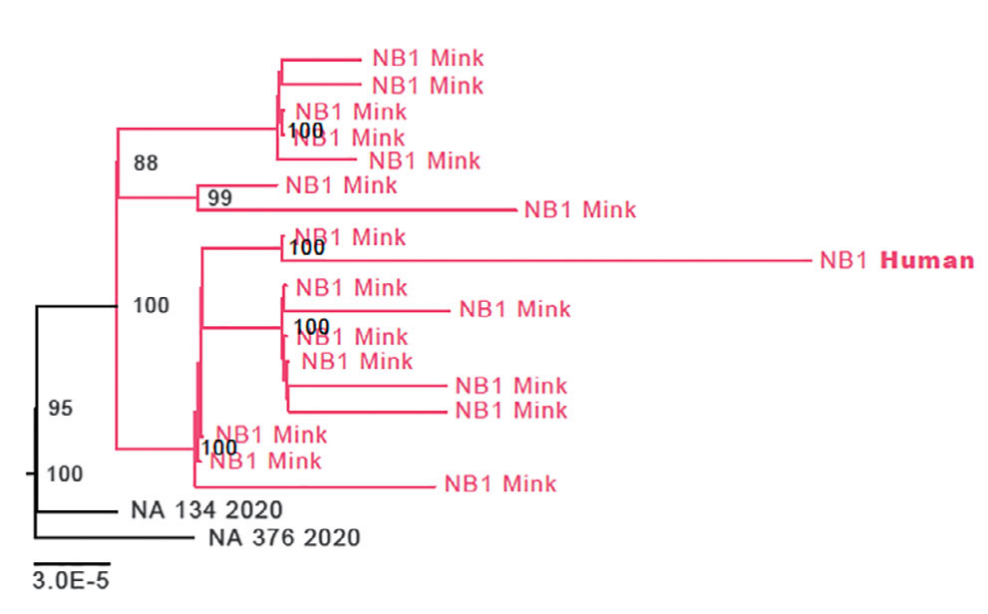
Phylogenetic analysis of mink farm NB1. A maximum likelihood analysis was performed using all available SARS-CoV-2 sequences from the Netherlands. Sequences from NB1 are depicted in red, and the employee of NB1 is shown in bold. The two sequences in black at the root of the cluster are the closest-matching human genome sequences from the national SARS-CoV-2 sequence database. The scale bar represents units of substitutions per site.

**Fig. 2:**
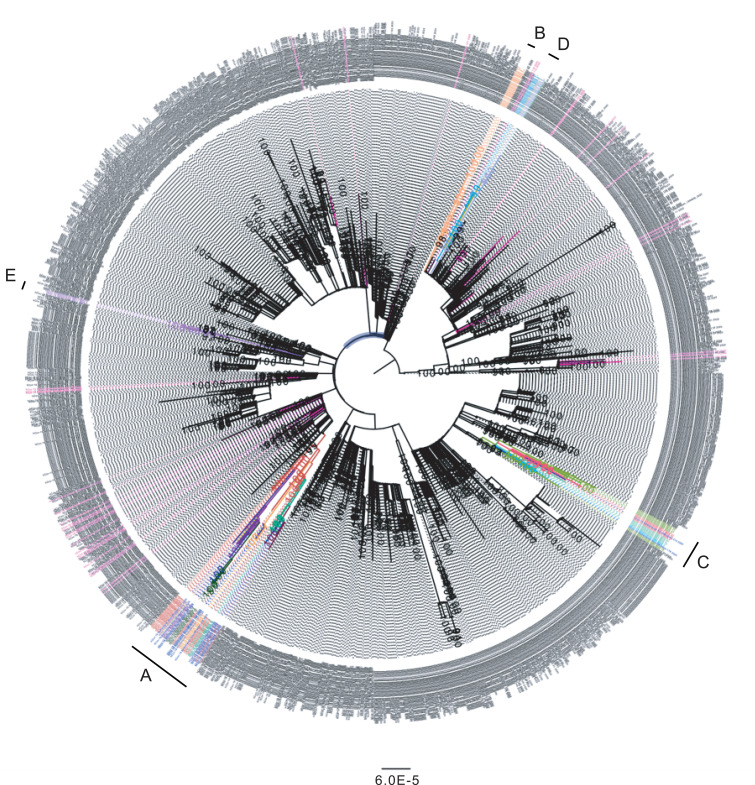
Maximum likelihood analysis of all SARS-CoV-2 sequences from the Netherlands. Sequences derived from minks from different farms are indicated with different colors, human sequences related to the mink farms are shown in blue, and samples from similar areas (as determined by four-digit postal code) are shown in magenta. The scale bar represents units of substitutions per site.

**Fig. 3:**
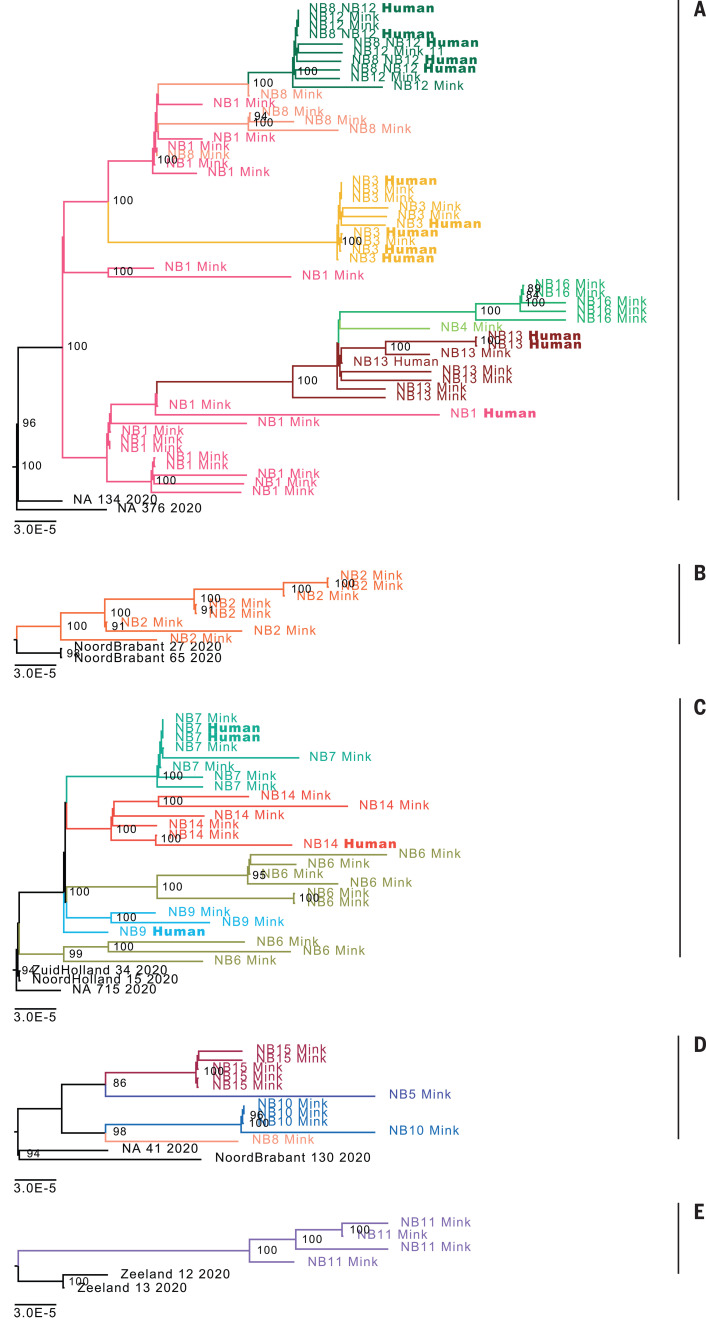
Phylogenetic analysis of 88 mink and 18 mink-related human SARS-CoV-2 sequences detected in the five mink farm clusters. Sequences derived from different farms are depicted in different colors. The scale bar represents units of substitutions per site.

During the interview on 28 April, four of five employees from NB1 reported that they had experienced respiratory symptoms before the outbreak was detected in minks, but none of them had been tested for SARS-CoV-2. Their symptom-onset dates ranged from 1 April to 9 May. For 16 of the mink (sampled on 28 April) and one farm employee (sampled on 4 May), a whole-genome sequence was obtained (hCov-19/Netherlands/NoordBrabant_177/2020). The human sequence clusters within the mink sequences, although it differs from the closest mink sequence by 7 nucleotides (nts) (Fig. 1 and cluster A in Figs. 2 and 3). On farm NB2, SARS-CoV-2 was diagnosed on 25 April. Retrospective analysis showed that one employee from NB2 had been hospitalized with SARS-CoV-2 on 31 March. All samples from the eight employees taken on 30 April were negative by RT-PCR but tested positive for SARS-CoV-2 antibodies. The virus sequence obtained from NB2 animals was distinct from that of NB1 animals, indicating a separate introduction (Figs. 2 and 3, cluster B).

On mink farm NB3, SARS-CoV-2 infection was diagnosed on 7 May. Initially, all seven employees tested negative for SARS-CoV-2, but when retested between 19 and 26 May after developing COVID-19–related symptoms, five of seven individuals working or living on the farm tested positive for SARS-CoV-2 RNA. Whole-genome sequences were obtained from these five individuals. The clustering of these sequences with the sequences derived from NB3 mink, together with initial negative test results and subsequent symptom onset, indicate that the employees were infected with SARS-CoV-2 after mink on the farm became infected. An additional infection was identified from contact tracing: An individual who did not visit the farm but had close contact with one of the employees became infected with the SARS-CoV-2 strain found on farm NB3. Animal and human sequences from farm NB3 were close to those from farm NB1, and both were categorized in cluster A.

Similarly, on mink farm NB7, zoonotic transmission from mink to humans likely occurred. On this farm, SARS-CoV-2 infection in mink was diagnosed on 31 May. NB7 employees initially tested negative for SARS-CoV-2, but several of them subsequently began to show symptoms. Between 10 June and 1 July, samples were taken from 10 employees, 8 of which tested positive for SARS-CoV-2 RNA. WGS of two NB7 employee samples showed that their virus sequences clustered with the sequences from the mink at this farm.

The sequences generated from animals and employees of mink farms were compared with ~1775 whole-genome sequences in the national database. To discriminate between community-acquired and mink farm–related infections and to determine the potential risk for people who live near mink farms, WGS was also performed on 34 SARS-CoV-2–positive samples (collected from 4 March until 29 April 2020) from individuals who live in the same geographic area (as determined by four-digit postal code) in which farms NB1 to NB4 are located. These local sequences, sampled in a proxy of ~19 km^2^, reflected the general diversity of SARS-CoV-2 seen in the Netherlands and were not related to the clusters of mink sequences found on the mink farms, thereby indicating that no spillover to people living in close proximity to mink farms had occurred and that the sequences from SARS-CoV-2–infected animals and farm workers clustered by farm (Fig. 2; sequences from community shown in magenta). The sequences from the mink farm investigation were also compared with sequences from Poland (*n* = 65) because many of the mink farm workers were seasonal migrants from Poland, but the Polish sequences were more divergent.

**Fig. 4:**
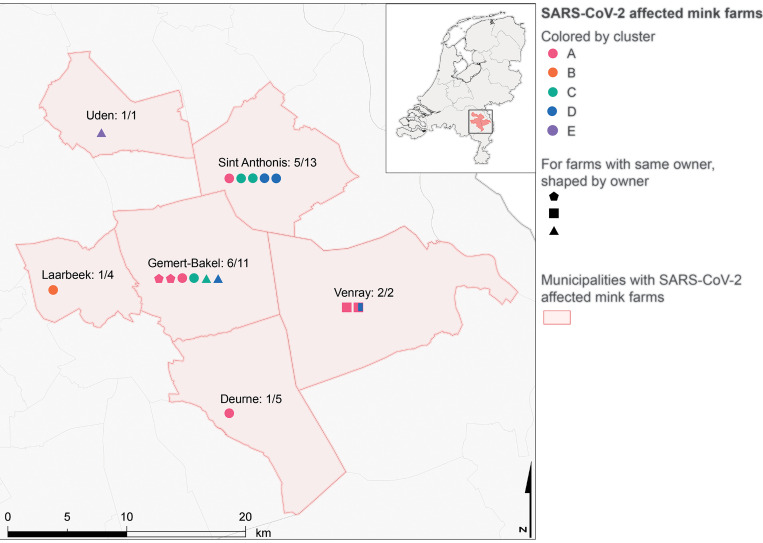
Geographic overview of mink farms with SARS-CoV-2–positive cases per affected municipality. The proportion of SARS-CoV-2–affected mink farms over the total number of mink farms (Central Bureau of Statistics database, 2019) is indicated for each area. Symbols for farms with positive cases are colored by cluster; non-circular shapes indicate farms with a single owner.

Phylogenetic analysis of the mink SARS-CoV-2 genomes showed that mink sequences of 16 farms were grouped into five different clusters (Figs. 2 and 3). Viruses from farms NB1, NB3, NB4, NB8, NB12, NB13, and NB16 belonged to cluster A; sequences from NB2 formed a distinct cluster (cluster B); those from farms NB6, NB7, NB9, and NB14 formed cluster C; those from NB5, NB8, NB10, and NB15 formed cluster D; and those from NB11 were designated as cluster E. On farm NB8, SARS-CoV-2 viruses from clusters A and D were found. A detailed inventory of possible common characteristics—including farm owner, shared personnel, feed supplier, and veterinary service provider—was made. Multiple farms within a cluster shared the same owner; however, in most cases no common factor could be identified for most farms, and clustering could not be explained by geographic distance (Table 2 and Fig. 4).

**Table 2:** Overview of the clusters detected on the different farms. Note that veterinarians II and V were from the same veterinary practice. In regard to detection, notification was based on reporting of clinical signs, which was required from 26 April onward. EWS-Ser detection was based on a one-time nationwide compulsory serological screening of all mink farms at the end of May or early June by GD Animal Health. EWS-PM detection was based on the early warning monitoring system (EWS) for which carcasses of animals that died of natural causes were submitted weekly for PCR testing by GD Animal Health from the end of May onward [EWS-PM-1st to -6th postmortem (PM) screening]. NA, not applicable.

**Farm**	**Date of** **diagnosis**	**Sequence** **cluster**	**Same** **owner**	**Feed** **supplier**	**Vet**	**No. of mink** **sequences (+no. of** **human sequences)**	**SNP differences** **(average)**	**Mink** **population** **size**	**Detection**
NB1	24 April 2020	A	NB1, NB4	1	I	17 (+1)	0 to 9 (3.9)	75,711	Notification
NB2	25 April 2020	B	NA	1	II	8	0 to 8 (3.6)	50,473	Notification
NB3	7 May 2020	A	NA	2	III	5 (+5)	0 to 2 (0.6)	12,400	Notification
NB4	7 May 2020	A	NB1, NB4	1	I	1	NA	67,945	Contact tracing NB1
NB5	31 May 2020	D	NA	1	IV	1	NA	38,936	EWS-Ser+PM-1st
NB6	31 May 2020	C	NA	3	V	9	0 to 12 (6.8)	54,515	EWS-Ser+PM-1st
NB7	31 May 2020	C	NB7, NB11, NB15	3	II	6 (+2)	0 to 4 (1.4)	79,355	EWS-PM-1st
NB8	2 June 2020	A and D	NB8, NB12*	3	V	6 (+5)	0 to 6 (2.6)	39,144	EWS-Ser+PM-1st
NB9	4 June 2020	C	NA	2	V	2 (+1)	0 to 3 (1.5)	32,557	EWS-Ser+PM-2nd
NB10	8 June 2020	D	NA	3	II	4	0 to 3 (1.1)	26,824	EWS-Ser+PM-2nd
NB11	8 June 2020	E	NB7, NB11, NB15	3	II	4	0 to 4 (2.2)	38,745	EWS-PM-2nd
NB12	9 June 2020	A	NB8, NB12*	3	II	5	0 to 3 (1.2)	55,352	Notification
NB13	14 June 2020	A	NA	3	V	5 (+3)	0 to 5 (3.2)	20,366	EWS-PM-5th
NB14	14 June 2020	C	NA	3	II	5 (+1)	0 to 7 (3.7)	28,375	EWS-PM-5th
NB15	21 June 2020	D	NB7, NB11, NB15	3	II	5	0 to 2 (0.6)	35,928	EWS-PM-6th
NB16	21 June 2020	A	NA	3	II	5	0 to 4 (1.6)	66,920	EWS-PM-6th

*There was exchange of personnel in these two locations.

In total, 18 sequences from mink farm employees or their close contacts were generated from seven different farms. In most cases, these human sequences were nearly identical to the mink sequences from the same farm. For NB1, the situation was different: The human sequence clusters deeply within the sequences derived from mink (Fig. 1), differing from the closest related mink sequence by 7 nts. This was also the case on farm NB14, with a 4-nt difference from the closest related mink sequence. Sequences of employees at mink farm NB8 clustered with those of animals from NB12, likely because personnel were exchanged between these two farms.

SARS-CoV-2 was detected on mink farms NB1 to NB4 after reports of respiratory symptoms and increased mortality in mink. The sequences from farm NB1 showed a difference of 0 to 9 single-nucleotide polymorphisms (SNPs) (average of 3.9 nts) and sequences from NB2 had a difference of 0 to 8 SNPs (average of 3.6 nts), which is more than is generally observed in outbreaks in human settings. In addition, two deletions (one of 12 nts and one of 134 nts) were observed in a single sequence from NB1. The sequences of mink at NB6 had differences of 0 to 12 SNPs, and a deletion of 9 nts was observed in one sequence, whereas diversity was lower for the subsequent farm sequences (Table 2). After the initial detection of SARS-CoV-2, farms were screened weekly. The first, second, fifth, and sixth weekly screening yielded new positive cases.

Several nonsynonymous mutations were identified among the mink sequences when compared with the Wuhan reference sequence NC_045512.2. However, no particular amino acid substitutions were found in all mink samples (fig. S1). Of note, three clusters had the position 614G variant (clusters A, C, and E), and two had the original variant. On the basis of the data available at this stage, there were no obvious disease presentation differences in animals or humans between clusters, but data collection and analysis are ongoing for cases after NB16. This D614G mutation that we observed can also be found in the general human population, and the same mutation was found in human cases related to the mink farms.

Our work shows evidence of ongoing SARS-CoV-2 transmission in mink farms and spillover events to humans. More research in minks and other mustelid species will be needed to determine whether these species are at risk of becoming reservoirs of SARS-CoV-2. After the detection of SARS-CoV-2 on mink farms, 68% of the tested farm workers and/or relatives or contacts would later become or had been infected with SARS-CoV-2, indicating that contact with SARS-CoV-2–infected mink is a risk factor for contracting COVID-19. Recently, an eightfold increase in cytidine-to-uridine (C→U) substitutions compared with uridine-to-cytidine (U→C) substitutions was described, suggestive of host adaptation (*41*). In the mink sequences, we observed a 3.5-fold increase in C→U compared with U→C substitutions, but the number of substitutions (185) was limited.

A high diversity was observed in the sequences from some mink farms, which is likely explained by multiple generations of viral infections in animals before the increase in mortality was detected. Current estimates indicate that the substitution rate of SARS-CoV-2 is around 1.16 × 10^−3^ substitutions per site per year in the human population (*42*), which corresponds to around one mutation per 2 weeks. This could mean that the virus was already circulating in mink farms for some time before it was identified. However, a relatively high sequence diversity was also observed on farms where dead animals tested under the early warning surveillance system tested negative 1 week before a positive test, hinting toward a faster evolutionary rate of the virus in the mink population. Mink farms have large populations of animals, living at high density, which may promote virus transmission. However, the moment of viral introduction was not known, which makes it difficult to draw definite conclusions about the substitution rate in mink farms. The generation interval for SARS-CoV-2 in humans has been estimated to be around 4 to 5 days (*43*), but with high-dose exposure in a farm with a high number and density of animals, this interval could potentially be shorter.

Further evidence that animals were the most likely source of human infection was provided by the clear phylogenetic separation between mink farm–related human and animal sequences and sequences from human cases within the same geographic area (as determined by four-digit postal code). However, some of the farm-related humans may have been infected within their household, not directly from mink. Spillback into the local community was not observed in our sequence data.

Thus far, the investigation has failed to identify common factors that might explain farm-to-farm spread, possibly via temporary workers who were not included in testing. Since our observations were made, SARS-CoV-2 infections have also been described in mink farms elsewhere (*44*–*46*). Additional research efforts are needed, as it is imperative that the fur production and trading sector should not become a reservoir for future spillover of SARS-CoV-2 to humans.

## Supplementary Materials

science.sciencemag.org/content/371/6525/172/suppl/DC1 Materials and Methods Fig. S1 Table S1 References (*47*–*63*) MDAR Reproducibility Checklist

## Appendix

View/request a protocol for this paper from *Bio-protocol*.
